# Expectations of outcomes in patients with colorectal cancer

**DOI:** 10.1002/bjs5.73

**Published:** 2018-05-10

**Authors:** A. L. Young, E. Lee, K. Absolom, H. Baxter, C. Christophi, J. P. A. Lodge, A. G. Glaser, G. J. Toogood

**Affiliations:** ^1^ Department of Hepatobiliary and Transplant Surgery St James's University Hospital Leeds UK; ^2^ Leeds Institute of Cancer and Pathology University of Leeds Leeds UK; ^3^ Department of Hepatobiliary and Transplant Surgery Austin Health Melbourne, Victoria Australia; ^4^ Austin Health Sciences Library Austin Health Melbourne, Victoria Australia

## Abstract

**Background:**

Understanding patients' expectations of their treatment is critical to ensure appropriate treatment decisions, and to explore how expectations influence coping, quality of life and well‐being. This study aimed to examine these issues related to treatment in patients with colorectal cancer.

**Methods:**

A literature search from January 1946 to September 2016 was performed to identify available data regarding patients' expectations of outcomes following colorectal cancer treatment. A narrative synthesis of the evidence was planned.

**Results:**

Of 4337 items initially identified, 20 articles were included in the review. In studies presenting data on overall and short‐term survival, patients considerably overestimated prognosis. Patients also had unrealistic expectations of the negative aspects of chemotherapy and stomas. There was marked discordance between patients' and clinicians' expectations regarding chemotherapy, end‐of‐life care, bowel function and psychosocial outcomes. Level of education was the most consistent factor influencing the accuracy of patients' expectations.

**Conclusion:**

Patients with colorectal cancer frequently have unrealistic expectations of treatment. Marked disparities exist between patients' and clinicians' expectations of outcomes.

## Introduction

Most patients with colorectal cancer are now expected to survive for 10 years or more as a result of earlier diagnosis and increasingly effective treatments^1^. Recommendations about treatment require both clinicians and patients to share an understanding of prognosis, management options and the goals of care^2^. Evaluating patients' expectations and using this information to guide treatment decision‐making is essential to achieve optimal outcomes^3^. Asking about expectations may lead to improved engagement in care and more realistic expectations of outcomes^4^. Unrealistic expectations may impair patients' ability to cope with the consequences of treatment, resulting in impaired quality of life. To date, little work has been done to explore the expectations of patients and clinicians regarding outcomes following treatment.

Systematic reviews typically address a specific question; however, scoping reviews are used widely to provide a map of the existing evidence on a broader topic in order to identify gaps in the knowledge base and identify areas for further study^5^, ^6^, ^7^. They use less strict inclusion criteria, bringing together data from more heterogeneous sources and, unlike systematic reviews which seek the best evidence, scoping reviews include all available evidence and so quality assessments are rarely performed^5^. The aim of this review was to assess studies reporting patients' expectations of treatment outcomes for colorectal cancer.

## Methods

### Search strategy

A preliminary search was conducted in the PubMed and Embase databases to inform the development of a set of keywords, index terms and phrases. These included: patient experience, perception or decision; informed decision or survival expectation; prognostic understanding, patient–physician communication or goals of care. Using these agreed terms, a second comprehensive search (*Appendix S1*, supporting information) was then conducted in Ovid MEDLINE (1 January 1946 to 14 September 2016), Ovid Embase (1 January 1974 to 14 September 2016), Ovid PsycINFO (1 January 1806 to 14 September 2016) and EBSCO CINAHL (1 January 1981 to 14 September 2016). The results were exported to reference management software, duplicates removed, and articles screened according to inclusion and exclusion criteria. The search strategy was supplemented by manual searches of bibliographic references. Grey literature was searched using Google Advanced and OpenGrey.

### Eligibility

Articles were included if primary data were presented on adult patients with colorectal cancer, and patient expectations regarding prognosis or effects of an intervention were assessed in any qualitative or quantitative manner. Articles that were not published in English were excluded. There was no restriction on study design, but articles had to be published in peer‐reviewed journals.

### Screening

After removal of duplicates, one author independently screened the remaining titles and abstracts, and extracted those that did not obviously meet the inclusion criteria. This list was checked by a second author. A full‐text screen of the remaining articles was carried out against the inclusion criteria. Any disagreements were resolved through discussion with the senior author.

### Data extraction and synthesis

The first author extracted data from the included studies and the second author reviewed the data for accuracy. A wide variety of outcomes can be assessed following colorectal cancer treatment^8^. The present review considered any article aimed to evaluate any outcome that had been assessed. Given the heterogeneity in patient groups, study design and data presented, a narrative synthesis was conducted of the findings from included studies. The results focused on the key areas in the available studies, including: expectations of overall survival, the impact of chemotherapy, psychosocial outcomes, end‐of‐life care and bowel function.

## Results

### Search results

**Figure 1:**
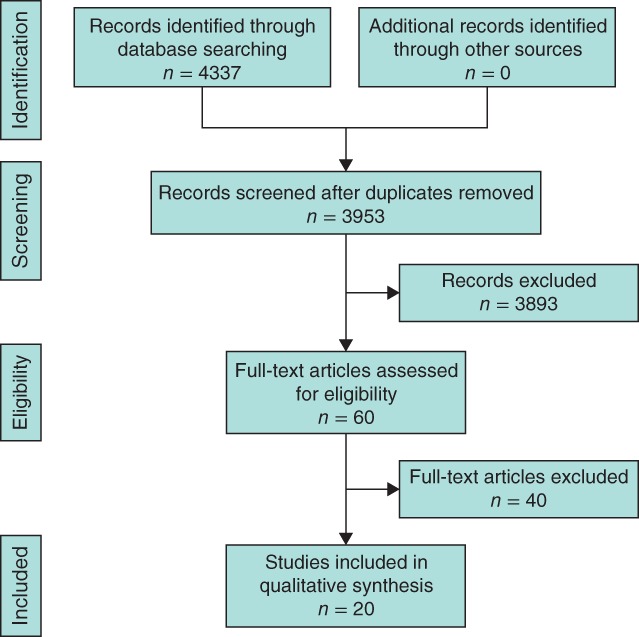
PRISMA diagram showing selection of articles for review

Of 4337 potential articles, 20 studies^9^, ^10^, ^11^, ^12^, ^13^, ^14^, ^15^, ^16^, ^17^, ^18^, ^19^, ^20^, ^21^, ^22^, ^23^, ^24^, ^25^, ^26^, ^27^, ^28^ were finally included in the review (*Fig*. 1). These studies are summarized in *Table* 1. The majority of articles came from North America (10 from the USA, 2 from Canada). Three were from Australia, and the remaining five from different European countries (Germany (2), the Netherlands, Italy and Austria).

**Table 1:** Summary of 20 studies included in the final review

Reference	Main aim	No. of patients with CRC	Group	Key findings	Methodology	Timing
Kim *et al*.^9^	To identify factors associated with patient perception of cure	2755	All stages	89.6% felt cure was likely; 79.6% with metastatic CRC felt cure was likely. Factors associated with decreased likelihood of cure were female sex, age > 75 years, being unmarried and white ethnicity	In‐house survey + validated questionnaires by mail	4–6 months after diagnosis
Mack *et al*.^10^	To assess how patient perceptions may influence care decisions	245	Stage IV only	Only one‐third of patients recognized that chemotherapy was not likely to cure them. This did not affect the likelihood of receiving chemotherapy but did influence the likelihood of receiving hospice care	In‐house survey by mail	4–6 months after diagnosis
Liu *et al*.^11^	To assess whether patients of physicians who discuss prognosis early have a more accurate awareness of life expectancy	295	Stage IV only	83.5% of patients did not have an accurate perception of prognosis. Patients of physicians who discuss prognosis early were more likely to have an accurate expectation	In‐house survey by mail	4–6 months after diagnosis
Park *et al*.^12^	To assess patient expectations of bowel function after rectal surgery and what influences these	26	All stages	A large degree of uncertainty. Patient attitudes play a key role in shaping expectations	Semistructured telephone interview by researcher	Before treatment
Mende *et al*.^13^	To assess patient perspective on palliative chemotherapy, and compare expected with actual survival	58	Palliative	A median survival of 3 years would be worth chemotherapy side‐effects. Patients on chemotherapy expected a median of 44 months, but achieved a median survival of 30 months	In‐house + validated questionnaires	Following chemotherapy
Zafar *et al*.^14^	To assess what influences patient decision‐making for chemotherapy	702	Stage IV only	Patients who felt chemotherapy was more likely to extend life were most likely to receive it	In‐house survey by mail	4–6 months after diagnosis
Neuman *et al*.^15^	To assess patient expectations and the impact of a temporary stoma	60	Patients with a stoma	Patients' perception of quality of life shifted over time	Qualitative telephone interview	3 time points before, during, after
Weeks *et al*.^16^	To assess outcome expectations of patients with metastatic CRC	1274	Palliative	81% of patients with metastatic CRC felt their palliative chemotherapy was going to be curative	Professional interviewers, computer‐assisted	4–7 months after diagnosis
Leighl *et al*.^17^	To assess the impact of decision aids on patients' understanding of options	207	Stage IV only	Most patients uncertain about prognosis, but 75% understand the impact of chemotherapy side‐effects	Survey	At treatment consultation and at 4 weeks
Emery^18^	To analyse patients' expectations of chemotherapy	30	Primary only	Patients believe that chemotherapy has to hurt to have benefit	Semistructured interviews	During/shortly after chemotherapy
Bossema *et al*.^19^	To assess patients' preference for surgical choice based on expectation of bowel function	122	Primary only	Patients will accept a high incontinence risk and even the loss of survival if this avoids a stoma	Semistructured interview + questionnaires	Following bowel surgery
Harrison *et al*.^20^	To assess patients' and clinicians' preferences for treatment options	103	Primary only	Clinicians and patients have different priorities based on different expectations. Patients are most keen to avoid the need for a stoma and radiotherapy	In‐house survey	Within a few days of surgery
Mastroianni *et al*.^21^	Crossover study comparing patients' expectations and experiences of oral and intravenous chemotherapy	20	Stage IV only	Patients prefer oral chemotherapy before any experience, but intravenous following experience of chemotherapy. Patient education level influences expectations	In‐house survey	Before and after treatment
Siassi *et al*.^22^	To assess expectations of patients before and after closure of a temporary stoma	35	Patients with a stoma	Patients tolerated stomas better than expected, but felt worse after stomal reversal than expected	Validated questionnaires + semistructured interview	4 weeks after treatment
Holzer *et al*.^23^	To assess preoperative expectations of outcomes following CRC surgery	167	Curative intent	Expectations are influenced by age, sex and level of education	In‐house survey	Before surgery
Salkeld *et al*.^24^	To assess the importance patients attach to various aspects of their treatment, outcomes and decision‐making	175	Primary only	Patients place trust in their surgeon as of the utmost importance in decision‐making. Cure is their main outcome priority	Trained interviewer + DCE survey	Immediately following primary treatment
Solomon *et al*.^25^	To investigate what aspects of quality of life patients are prepared to trade off against survival	100	Curative intent	Patients were willing to gamble survival on avoiding a stoma or chemotherapy. There were marked differences between clinicians' and patients' expectations of outcomes	Structured interview	Inpatients
McCarthy *et al*.^26^	To assess patients' care preferences in end‐of‐life care	520	Palliative	There was a marked disparity between patients' and clinicians' expectations of outcome. Patients favoured comfort measures more as death approaches	Interviews	During palliative care
Haidet *et al*.^27^	To assess expectation of prognosis in 2 and 6 months' time	520	Palliative	Patients overestimated prognosis, but had good quality of life until late‐stage disease	Trained interviewers	During palliative care
Weeks *et al*.^28^	To assess patients' perception of outcome and concordance with clinicians	362	Palliative	Patients were more optimistic than clinicians; clinicians were more accurate. Patients' expectations influenced the choice of treatment	Interviews	During palliative care

CRC, colorectal cancer; DCE, discrete choice experiment.

Thirteen studies investigated only patients with colorectal cancer, whereas seven also involved patients with other cancers, predominantly lung (6 of 7 studies). Ten studies concentrated on patients with end‐stage disease, four focused on those having primary surgery only, four assessed patients with a range of stages and two looked specifically at patients with temporary stomas. In 12 studies, a variety of questionnaires were used (almost entirely in‐house questionnaires) and nine included semistructured interviews. The median study population was 171 (range 20–2755) patients. Treating clinicians' expectations of patients' outcomes were also evaluated in five studies.

Only two studies were longitudinal. One^17^ was primarily concerned with assessing the benefits of a decision aid, and the other^15^ on the impact of a stoma and stoma reversal. There was one crossover study (patients receiving oral then intravenous chemotherapy or the reverse)^21^. The other 17 studies were cross‐sectional.

### Overall survival

*Table* 2 summarizes the five studies that had data on patients' expectation of survival. Only one study^13^ compared expectation of survival with actual survival. Patients achieved a median survival of 30 months, but expected a median of 44 months. Three studies^26^, ^27^, ^28^ evaluated expectations regarding the probability of surviving the next 2 or 6 months among patients being treated with palliative intent. In each of these studies, patients again overestimated their probability of survival. It was found that their expectation may have a significant impact on the treatment they received. Patients with the longest expectation of survival were more likely to receive potentially life‐extending therapy rather than simply supportive care^28^.

**Table 2:** Survival expectations

Reference	Findings
Kim *et al*.^9^	95.3% of all patients felt surgery would prolong life; 89.6% felt it would cure them; 45% felt surgery would be accompanied by complications; 79.6% of patients with metastatic CRC felt surgery was likely to cure them
Mack *et al*.^10^	Only one‐third of the patients studied who received chemotherapy in the last month of their life recognized that it would not cure them
Liu *et al*.^11^	86% of patients did not have an accurate expectation of their prognosis. Patients of physicians with larger numbers of terminally ill patients, patients of physicians who discuss prognosis early, and those closest to death were more likely to have a more accurate expectation
Mende *et al*.^13^	Patients being treated with palliative chemotherapy expected a median survival of 44 months; median actual survival was 30 months
Weeks *et al*.^16^	81% of patients with metastatic CRC undertaking palliative chemotherapy felt their treatment was likely to be curative. Patients with accurate expectations were more likely to be of white ethnicity, from an integrated health network and to grade communication received as poor

CRC, colorectal cancer.

In all studies that compared patients' and clinicians' estimates of prognosis^13^ ^20^, ^25^ ^26^, ^28^, the clinicians had a more accurate expectation of prognosis. This was quantified in only one study^27^, which showed the area under a curve for a physician's estimation of prognosis to be 0·80, compared with 0·66 for a patient.

### Chemotherapy

Eight studies examined patients' expectations of the impact of chemotherapy (*Table* 3). They approached patient expectation from a variety of viewpoints, including investigating the perceptions of beneficial effects, burden of side‐effects and preference for route of administration. Patients tended to overestimate the beneficial effects of treatment. They may also have had unrealistic expectations regarding side‐effects. Patients receiving chemotherapy frequently thought that ‘it's got to hurt to have benefit’^18^. One study^17^ showed that patients' preconceived ideas regarding chemotherapy before a consultation significantly influenced their likelihood of uptake, and a further study^25^ found that some were willing to potentially limit longevity owing to concerns about the side‐effects of chemotherapy. Following personal experience of chemotherapy, patients' perspectives on choosing methods of administration often changed^21^. Higher education level significantly influenced patient decision‐making both in terms of having more realistic expectations and improved decisional certainty^17^ ^21^.

**Table 3:** Expectations of chemotherapy

Reference	Findings
Mack *et al*.^10^	One‐third of patients recognized that chemotherapy offered no chance of cure
Mende *et al*.^13^	Patients felt a median threshold survival of 36 months was required to benefit from palliative chemotherapy. Patients expected a median survival of 44 months; 30 months was achieved, although trial data would have anticipated a median of 19 months
Zafar *et al*.^14^	Patients who wanted to prolong life were more likely to receive chemotherapy than those who focused on comfort. Patients who thought chemotherapy would extend their life were more likely to receive chemotherapy than those who thought this would be unlikely
Weeks *et al*.^28^	81% of patients had inaccurate expectations of the beneficial effects of chemotherapy
Leighl *et al*.^17^	Patients were more likely to want chemotherapy following a consultation if they had more knowledge or if they had a preconceived opinion about wanting chemotherapy before the consultation
Emery^18^	Patients felt chemotherapy had to hurt and have significant side‐effects to have a beneficial effect, and that intravenous was more powerful than oral administration
Mastroianni *et al*.^21^	There was a correlation between education level and preference for oral or intravenous chemotherapy. Preferences regarding chemotherapy changed after receiving chemotherapy, with the side‐effect profile a more important factor
Solomon *et al*.^25^	Patients had a greater reluctance to have chemotherapy than surgery with a stoma and were willing to gamble survival time to avoid chemotherapy

### Quality of life

Only two studies^19^ ^23^ attempted to evaluate quality‐of‐life expectations. Using an in‐house questionnaire, one^23^ found that age and level of education significantly influenced patient expectations of outcomes before treatment. All groups in that study gave highest priority to achieving cure, although older patients less frequently expected cure than younger patients. Younger patients were also more likely to expect to avoid a stoma and were more concerned by the prospect of incontinence^23^. Cosmetic issues, quick return to work, ability to attend social events and an undisturbed sex life were all expected significantly more often among younger patients^23^.

The other study^19^ investigating quality of life used its own questionnaires as well as the EuroQoL Five Dimensions (EQ‐5D™; EuroQol Group, Rotterdam, the Netherlands), although the primary aim was to investigate the patient's perception of likelihood of cure and the influence of physician communication. Some 83 per cent of patients felt surgery was likely or very likely to help with ‘some of the problems’ they were currently experiencing as a result of their cancer, and 95 per cent felt it was likely to prolong life^19^.

### End‐of‐life management

Ten studies included patients with advanced colorectal cancer, of which five addressed end‐of‐life care. One study^26^ that looked mainly at patients in their last 6 months of life showed how treatment preferences changed as individuals got closer to death, with an increasing focus on comfort rather than longevity. Patients were less keen to prolong life with care options such as the requirement for a feeding tube and there was an increasing preference for ‘do not resuscitate’ orders to be applied. This latter finding was confirmed in a second study^27^, while highlighting that physicians incorrectly overestimated preferences not to be resuscitated in one‐third of patients^27^.

### Bowel function

Two studies specifically addressed patients' attitudes to a temporary stoma, with questionnaires delivered before and at varying time points following the procedure. One study^15^ looked at patients' preoperative expectations of the effect of a temporary stoma. The authors identified a response shift in perception of quality of life that they attributed to the life‐threatening disease and the time spent living with a temporary stoma. In a further study^22^, notwithstanding a lack of clarity around the timing of questionnaires and interviews, some 55 per cent of patients felt the experience of living with a stoma positively exceeded their expectations and 30 per cent thought it met their expectations. After stoma reversal, however, 6 per cent of patients felt worse than expected, with expectations being exceeded in only 5 per cent and being met in 35 per cent. In two studies^21^ ^25^, patients were prepared to compromise survival to avoid a stoma. One study^25^ reported that patients were prepared to accept a high incontinence rate and even willing to compromise on survival in order to avoid a stoma, and a further study^21^ implied that 60 per cent of patients were prepared to give up one‐third of their life expectancy in order to avoid a stoma.

One study^12^ explored preoperative patient expectations of bowel function following rectal cancer surgery. Sources of patients' expectations and understanding, such as information from healthcare providers, were evaluated. A high degree of patient uncertainty and worry about future bowel function was present, with wide variations in individual patient views on how this might affect their lives. Bowel function problems, however, were often seen as a secondary issue compared with anxieties around cancer cure and recovery from major surgery.

## Discussion

Evaluating patients' expectations following colorectal cancer treatment involves considering both patients treated with curative and those treated with palliative intent. Understanding patients' expectations encompasses perceptions of prognosis as well as the physical consequences of cancer and proposed treatments. Although colorectal cancer is a common cancer, there is a paucity of literature on this topic. Although formal assessments of study quality have not been carried out, in line with typical scoping review methodology^5^, it is apparent that this area also lacks robust, longitudinal studies with validated questionnaires that may determine how patient expectations change over time and how they may change in response to treatments or the provision of information as their condition evolves. As the studies identified focused on a range of issues using a variety of study designs, a systematic review could not be performed.

Disparities between patients' and clinicians' expectations of prognosis were consistent findings. Of five studies^13^ ^20^, ^25^ ^26^, ^28^ that compared the two, all demonstrated marked differences in expectations. Many patients receiving chemotherapy with palliative intent actually felt the goal was still cure^17^. Similar disparity between patients' and clinicians' expectations was also demonstrated in ‘do not resuscitate’ decisions among those with end‐stage disease. Clinicians incorrectly estimated the patient's preference not to be resuscitated in one‐third of patients^27^. The impact of education level influencing patient expectations was touched on in several studies^21^ ^23^, but only one study^12^ evaluated how patients with colorectal cancer developed their expectations of outcome.

Patients' expectations have been shown to be a strong predictor of the success of surgical interventions^29^ ^30^, and in heart disease can even influence survival^31^. Modifying patients' expectations before cardiac surgery has resulted in less disability and better quality of life, earlier return to work, lower readmission rates and, interestingly, significantly reduced levels of the proinflammatory cytokines interleukin (IL) 6 and IL‐8^32^.

To evaluate patient outcomes accurately it is important to understand the patient's expectations before treatment, what is most important to the patient, and what their individual priorities and anxieties are. It is essential to make this assessment before the intervention as this will reduce the impact of cognitive dissonance, where the patient's beliefs and values may be recalibrated following the intervention^33^.

More high‐quality research needs to be done in this area. Better understanding is needed of patients' expectations of outcomes, their influence on treatment decisions, quality‐of‐life outcomes, and the variability in outcome expectations across disease stages and patient factors. Subsequently, interventions that realign patients' and clinicians' expectations to ensure shared decision‐making can be investigated, which will probably lead to improved outcomes.

## Disclosure

The authors declare no conflict of interest.

## Supporting information


**Appendix S1** Search strategy
