# Supplementary material for: Expectations of outcomes in patients with colorectal cancer
Source: BJS Open. 2018 May 10;2(5):285–92. doi: 10.1002/bjs5.73 (PMC6156162; doi:10.1002/bjs5.73)
Supplement: Supplementary file 1 — Appendix S1 Search strategy [file BJS5-2-285-s001.docx]

**Expectations of outcomes in patients with colorectal cancer**

A. L. Young, E. Lee, K. Absolom, H. Baxter, C. Christophi, J. P. A. Lodge, A. G. Glaser and G. J. Toogood

**Appendix S1** Search strategy

**MEDLINE** – Ovid platform, search conducted 14 Sep 2016

1. exp Colorectal Neoplasms/

2. (colonic neoplasm or colorectal neoplasm or rectal neoplasm or colorectal cancer or colorectal carcinoma or colorectal tumo$r or bowel cancer or bowel neoplasm or bowel carcinoma).tw.

3. (cancer adj3 (colon or rectum or bowel)).tw.

4. or/1-3

5. (survival adj2 (expectation* or prognosis)).tw.

6. (patient* adj2 (experience* or decision or decision making or expectation* or preference* or perception or communication or satisfaction or understanding or knowledge or belief* or participation or reported outcome or outcome assessment)).tw.

7. (shared adj2 decision making).tw.

8. or/5-7

9. (patient adj2 (provider communication or physician communication or physician engagement or doctor relationship)).tw.

10. (informed decision or survival discussion or standard gamble or prognostic understanding or expectation assessment or expectation discussion or prognosis discussion or information provision).tw.

11. ((communication or discussion) adj2 (treatment or procedure or surgery)).tw.

12. "goals of care".tw.

13. or/9-12

14. 8 or 13

15. 4 and 14
